# Fe_3_O_4_@SiO_2_@CSH^+^VO_3_^−^ as a novel recyclable heterogeneous catalyst with core–shell structure for oxidation of sulfides

**DOI:** 10.1038/s41598-024-58552-3

**Published:** 2024-04-08

**Authors:** Ula Zuhair Ismael Al-Zubaidi, Kiumars Bahrami, Minoo Khodamorady

**Affiliations:** 1 Department of Organic Chemistry, Faculty of Chemistry, Razi University, Kermanshah, 67144-14971 Iran; 2 Nanoscience and Nanotechnology Research Center (NNRC), Razi University, Kermanshah, 67144-14971 Iran

**Keywords:** Fe_3_O_4_ NPs, Chitosan, NH_4_VO_3_, H_2_O_2_, Sulfoxide, Catalysis, Green chemistry, Organic chemistry

## Abstract

Iron nanoparticles, with low toxicity and many active sites, are among the materials that not only reduce waste along with green chemistry but also increase the separation power and recover the catalyst from the reaction environment. In this study, first, the surface of iron nanoparticles was silanized, and in the next step, the complex of chitosan HCl.VO_3_ was placed on the surface of Fe_3_O_4_ (Fe_3_O_4_@SiO_2_@CSH^+^VO_3_^−^). This nanocatalyst is a novel, recoverable, and potent nanocatalyst with high selectivity for the oxidation of sulfides to sulfoxides. Various physicochemical techniques such as IR, XRD, TGA, SEM, EDX, mapping, TEM, and VSM were used to affirm the well synthesis of the catalyst. Oxidation of sulfides in the presence of hydrogen peroxide as a green oxidant and in ethanol was catalyzed by the Fe_3_O_4_@SiO_2_@CSH^+^VO_3_^−^. All sulfoxides were achieved with high efficiency and in a short time. The notable privileges of this method include facile and economic catalyst synthesis, proper catalyst durability, great performance, simple catalyst isolation, good recovery capability, at least up to 5 times without an index drop in catalytic power.

## Introduction

Despite their excellent activity and high selectivity, homogeneous catalysts have disadvantages such as difficult separation and lack of reusability. In recent decades, researchers have increasingly focused on heterogenizing homogeneous catalysts through various processes^1^. Heterogeneous catalysts do not have the problem of separation and recovery. Today, the stabilization of the homogeneous part of the catalyst on the surface of different solid substrates such as zeolite, MAF, boehmite, iron nanoparticles, polymers, etc. is one of the most common methods for heterogenization that has been reported^2–8^. Heterogeneous nanostructures possess many active sites, a high specific surface area, tunable physical properties, and excellent catalytic properties due to the presence of nano-sized particles.

Until now, various solid substrates such as SBA-15^9–11^, alumina^12^, Silica nanoparticles^13^, Fe_3_O_4_ NPs^14,15^, MCM-41^16^, polymers^17^, boehmite^18–20^, graphene oxide^21,22^, nano fibers^23^, CoFe_2_O_4_ ferrites^6^ have been used to heterogenize homogeneous catalysts. Some of these substrates, such as ionic liquids, polymers, and heteropoly acids, are expensive^24–26^. Among the solid supports used, iron nanoparticles are very popular as catalysts in synthetic chemistry due to their unique and numerous properties^27–29^. Among the iron oxides, Fe_3_O_4_ or magnetite is of interest to researchers because it has a high active surface, low toxicity, acceptable cost, good stability, and the ability to be easily separated from the environment with an external magnet^1,30,31^.

The synthesis of sulfoxides through the selective oxidation of sulfides using transition metals is considered one of the important and valuable reactions, both from industrial perspectives and in laboratory synthesis^32,33^. Sulfoxides serve as highly valuable structural frameworks in the synthesis of biologically and chemically active molecules, including flavors, drugs, germicides, and more^34^. By reviewing the scientific literature, one can highlight the importance of these compounds in enzyme activation, drug synthesis, agricultural chemistry, and their utility as solvents^35^. According to researchers, selective oxidation in the presence of safe, green, environmentally friendly, inexpensive, and highly efficient oxidants is of paramount importance. Thus far, a multitude of oxidants have been employed for the oxidation of sulfides, many of which are not only hazardous and toxic but also tend to produce over-oxidation products^36,37^. Today, in line with green chemistry principles, environmental compatibility, enhancing atom economy, and reducing over-oxidation, numerous research studies focusing on the use of hydrogen peroxide (H_2_O_2_) have been reported. H_2_O_2_ is a mild, cost-effective oxidizer with high oxygen content that produces only water as the sole byproduct^38,39^.

Chitosan is a polysaccharide polymer derived from waste generated in seafood processing industries. It is worth noting that chitosan ranks as the second most abundant biopolymer on earth  after cellulose. Chitosan is a polysaccharide polymer derived from waste generated in seafood processing industries. It is worth noting that chitosan ranks as the second most abundant biopolymer on earth  after cellulose^40^. Chitosan can be commercially obtained through acetylation of chitin in alkaline medium.

Vanadium is one of the transition metals in the third row of the periodic table, exhibiting various coordination numbers. It is abundantly present on the earth's  surface, surpassing the quantities of copper and palladium, and is associated with fewer adverse effects on the environment compared to many other metals^41–43^. Up to now, various catalytic systems based on transition metals and hydrogen peroxide have been explored for the conversion of sulfides to sulfoxides. Unfortunately, these systems suffer from disadvantages such as environmental hazards, complicated reaction procedures, long reaction times, low efficiency, and the use of expensive reagents^44^.

So far, heterogeneous catalysts such as: VMOP-8^45^, Zr_12_-NBC^46^, VO-TAPT-2,3-DHTA COF^47^, Ti(SO_4_)_2_@GOF^48^, Br_3_-Fe_3_O_4_^49^ Zr(IV)/imine@Fe_3_O_4_^50^, CuNPs/NC^51^ and FeL^GDC^-AP@GO^52^ have been reported for the oxidation of sulfides. Based on the materials mentioned, the need for the synthesis and design of heterogeneous nanocatalysts that are inexpensive, environmentally compatible, recoverable, stable, and highly active is consistently a concern among researchers. In this study, a novel nano-heterostructure was synthesized by placing chitosan complexed with VO_3_.HCl on the surface of silicified iron nanoparticles. This synthesized structure exhibited excellent activity and selectivity in converting sulfides to sulfoxides at room temperature in the presence of methanol and hydrogen peroxide, serving as a safe and environmentally friendly oxidant.

## Experimental

### Material and methods

The materials used in this study include FeCl_3_, NaOH, FeCl_2_, EtOH, CH_2_Cl_2_, tetraethyl orthosilicate (TEOS), chitosan (CS), HCl and NH_4_VO_3_, all of which were purchased from Merck without purification. The FT-IR spectra were recorded using a Shimadzu IR-470 spectrophotometer. TGA spectra were obtained using the STA504 device in the temperature range of 25–1000 °C, with the temperature increasing by 10 °C every minute during the analysis. Results from EDX-mapping analyzes were recorded with a Brucker TESCAN equipped with a SAMX Detector. FESEM images were acquired using a TESCAN MIRA3 at various magnifications. The magnetic strength of the catalyst was determined using a VSM apparatus from Magnatis Kavir Kashan Company. TEM images were captured using a CM 120 instrument from the Netherlands with a maximum voltage of 100 KV. X-ray diffraction patterns were prepared using a JEOL-JDX-8030 instrument (30 KV, 20 mA).

### Synthesis of Fe_3_O_4_ NPs

Iron nanoparticles were synthesized based on the method mentioned in scientific reports^15,19^.

### Silanization of the surface of Fe_3_O_4_ NPs

To silanize the surface of iron nanoparticles, 0.5 g of iron nanoparticles were dispersed in 25 mL of a mixture containing water and ethanol in a volume ratio of 1:8. Then, 1 mL of ammonia solution was added to the dispersion. After a few minutes, 1 mL of tetraethyl orthosilicate (TEOS) was added to the mixture, which was then stirred for 12 h at room temperature. Upon completion of the reaction time, the nanoparticles were collected using a strong magnet, washed with water and ethanol, and finally dried in an oven at 50 °C.

### Immobilization of chitosan hydrochloride on the surface of Fe_3_O_4_@SiO_2_

Chitosan hydrochloride was obtained by dissolving chitosan in 20 cc of a 1% hydrochloric acid solution under stirring at 1000 rpm. Subsequently, 0.5 g of silanized iron nanoparticles was added to this solution, and the mixture was refluxed for 24 h. The synthesized Fe_3_O_4_@SiO_2_@CS.HCl was easily collected using a strong magnet and rinsed at 60 °C.

### Formation of Fe_3_O_4_@SiO_2_@CSH^+^VO_3_^−^

**Figure 1:**
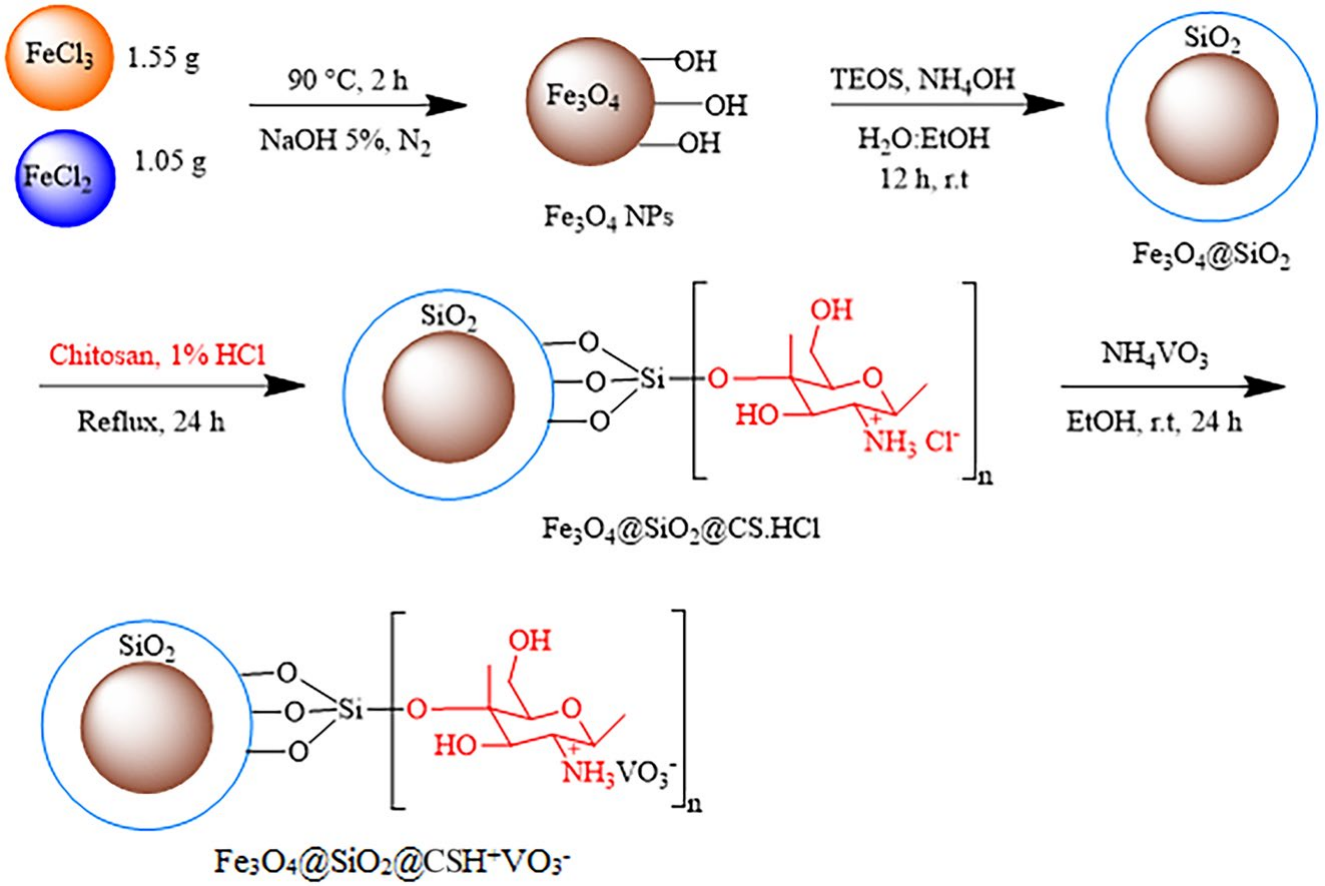
Catalyst synthesis steps.

To synthesize the final catalyst, 0.1 g of ammonium metavanadate (NH_4_VO_3_) was added to 0.5 g of Fe_3_O_4_@SiO_2_@CS.HCl nanoparticles in ethanol. The mixture was refluxed at room temperature for 24 h. After the desired time, the resulting nanoparticles were easily collected using a strong magnet and washed several times with ethanol and water. Finally, they were dried at 50 °C to produce Fe_3_O_4_@SiO_2_@CSH^+^VO_3_^−^ (Fig. 1).

### A general procedure for the synthesis of sulfoxides

**Figure 2:**
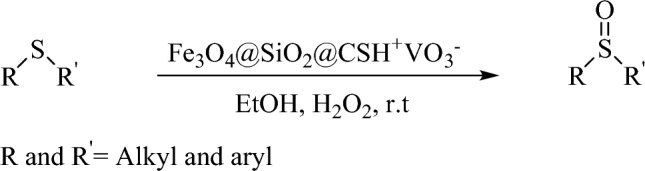
Oxidation of sulfides using Fe_3_O_4_@SiO_2_@CSH^+^VO_3_^−^

A mixture of 30% hydrogen peroxide (0.4 mL) and sulfide (1 mmol) was added to a round-bottom flask containing Fe_3_O_4_@SiO_2_@CSH^+^VO_3_^−^ (0.05 g). The resulting mixture was vigorously stirred in ethanol at ambient temperature. The progress of the reaction was monitored by TLC. Upon completion of the reaction, the nanocatalyst was easily removed using a powerful external magnet. The products were then extracted by adding water and ethyl acetate. After evaporation of the organic solvent, the desired sulfoxides were obtained with high purity (Fig. 2).

## Results and discussion

### Catalyst characterization

**Figure 3:**
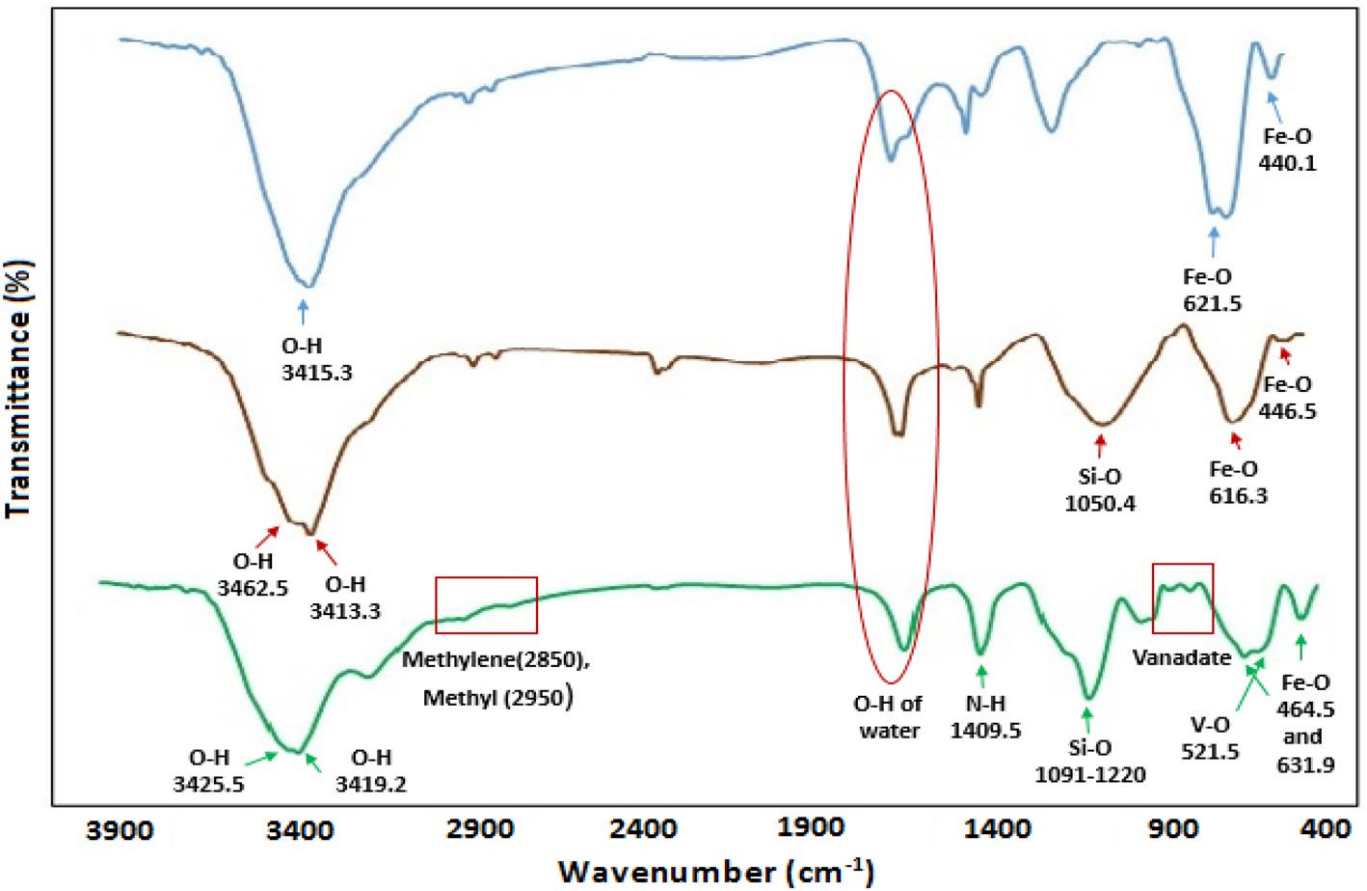
FT-IR curves of Fe_3_O_4_NPs (blue curve), Fe_3_O_4_@SiO_2_ (red curve), Fe_3_O_4_@SiO_2_@CSH^+^VO_3_^−^ (green curve).

Figure 3 shows the FT-IR curves of Fe_3_O_4_NPs (blue curve), Fe_3_O_4_@SiO_2_ (red curve), Fe_3_O_4_@SiO_2_@CSH^+^VO_3_^−^ (green curve). In blue curve, peaks appearing at 440.1 and 621.5 cm^−1^ can be attributed to Fe–O in cubic structure of Fe_3_O_4_ NPs. The stretching vibration of OH groups in iron nanoparticles appeared at 3415.3 cm^−1^. In all the curves, the bending vibration of OH (water) has appeared in the region of 1640–1645 cm^−1^. In the FT-IR of Fe_3_O_4_@SiO_2_ NPs (red curve), in addition to the peaks related to Fe–O in the range of 446.5–616.3 cm^−1^, the stretching vibration of Si–O has appeared at 1054.4 cm^−1^. Also, OH stretching vibrations related to TEOS (Tetraethyl orthosilicate) and Fe_3_O_4_ NPs are observed in 3413.3 and 3462.5 cm^−1^, respectively. In FT-IR of Fe_3_O_4_@SiO_2_@CSH^+^VO_3_^−^ (green curve), peaks appearing at 464.4 and 631.9 cm^−1^ indicate the presence of Fe–O in the nanocatalyst structure and the successful synthesis of iron nanoparticles^15,19^. The presence of V–O can be confirmed by the vibrational frequency at 521.5 cm^−1^, also three frequencies in the regions of 794.2, 851.4 and 935 are assigned to polymeric vanadate groups^53^. The two peaks appearing at 1091 to 1220 cm^−1^ are related to the Si–O bond, which indicates the synthesis of the core–shell structure of silanized Fe_3_O_4_@SiO_2_ NPs^54^. The presence of chitosan in the nanocatalyst structure is proved by the strong symmetric stretching frequency of the N–H group at 1409.5 cm^−1^^14,55^. Based on the Fig. 3 stretching absorptions of methylene and methyl groups appeared in the region of 2850 and 2940 cm^−1^. The vibration observed at 1629.6 cm^−1^ can be related to the bending frequency of the hydroxyl group in the structure of chitosan, water and Fe_3_O_4_ NPs^15,19,35^. The stretching vibrations related to the OH groups in iron nanoparticles appeared at 3419.2 cm^−1^ and the OH group of water molecule in the structure of nanocatalyst appeared at 3425.5 cm^−1^.

**Figure 4:**
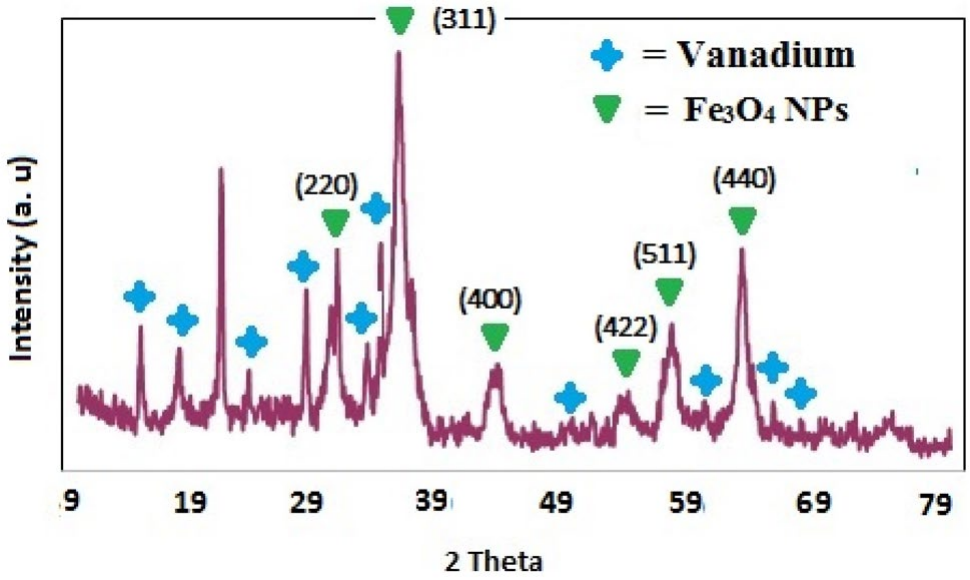
XRD graph of Fe_3_O_4_@SiO_2_@CSH^+^VO_3_^−^

X-ray diffraction technique was used to determine the crystalline structure of the synthesized nanocatalyst (Fig. 4). The peaks appearing at 2theta 15.21, 18.41, 23.86, 28.51, 33.46, 34.41, 49.86, 51.51, 60.51, 65.81, and 68.16 indicate the excellent binding of ammonium vanadate on the surface of silanized magnetic nanoparticles^56^. Diffraction peaks appearing at 30.41 (220), 35.51 (311), 43.91 (400), 53.91 (422), 57.71 (511) and 63.36 (440) confirms the cubic structure for iron nanoparticles (JCDPS card no, 19–0629)^57^. In order to obtain the size of the particles, the Debye Scherrer Eq. (1) was used. After calculations, the particle size was obtained in the range of 15 to 72 nm.

1 $$D = K\lambda /\beta \, Cos\theta$$

here, *D* is the crystallite size, *K* is the shape factor, calculated for spherical particles is 0.98, *K* = 1.54 A◦ for Cu and *β* is full width at half maxima of the highest peak in radian.

**Figure 5:**
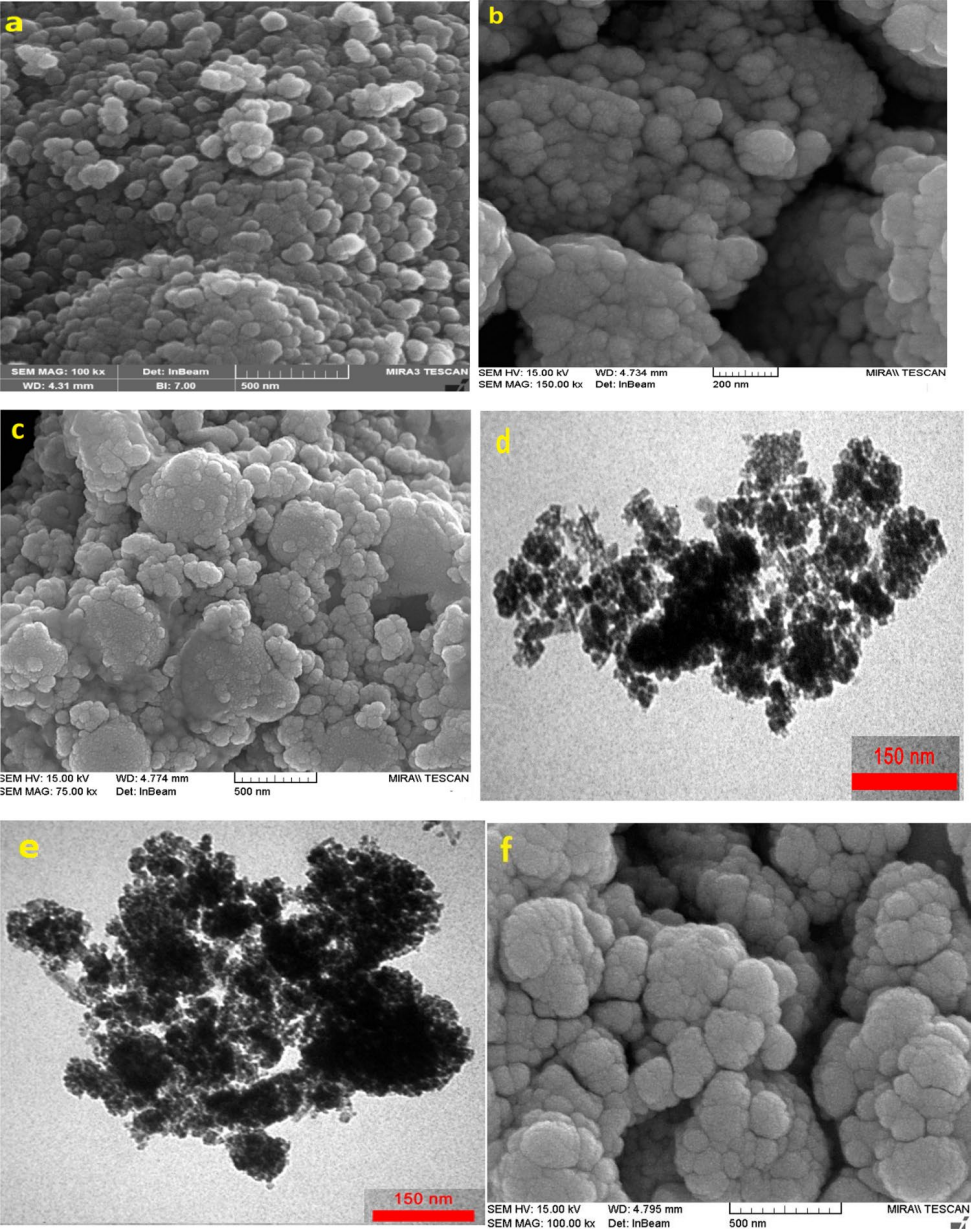

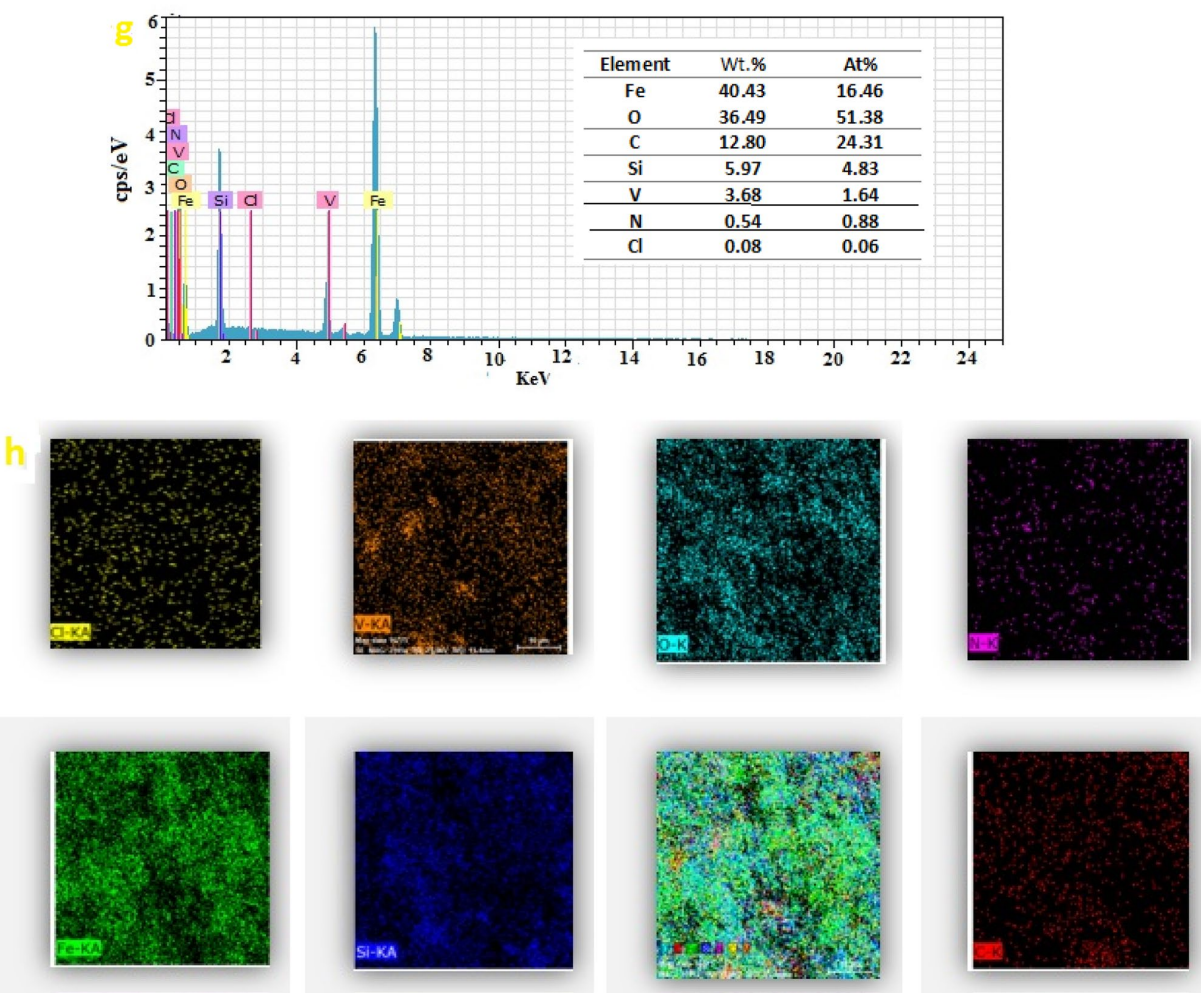
**(a)** FESEM picture of Fe_3_O_4_NPs, **(b)** FESEM image of Fe_3_O_4_@SiO_2_, **(c)** FESEM image of catalyst, **(d)** TEM picture for the Fe_3_O_4_@SiO_2_@CSH^+^VO_3_^−^, **(e)** TEM picture of catalyst after 5th use **(f)** FESEM image of catalyst after 5th run, **(g)** EDX pattern of Fe_3_O_4_@SiO_2_@CSH^+^VO_3_^−^, **(h)** Elemental Mapping of Fe_3_O_4_@SiO_2_@CSH^+^VO_3_^−^.

The morphology and shape of synthesized nanoparticles were scrutinized by FESEM analysis. FESEM illustrations of Fe_3_O_4_ NPs, silanized iron nanoparticles and heterogeneous nanocatalyst are shown in Fig. 5a–c. Nanoparticles in all photos are almost uniformly distributed and have a relatively spherical structure with a size of 40–80 nm. After binding the chitosan-HCl.VO_3_ complex on the surface of Fe_3_O_4_@SiO_2_, there was no change in the morphology of the nanoparticles. TEM analysis was used to acknowledge the core–shell shape of magnetic nanocatalyst and estimate the exact size of the particles. Figure 5d reveals the core–shell structure of nanoparticles and the average particle size is betwixt 30–40 nm. Figure 5e displays the TEM image after 5^th^ use of the catalyst. Based on this picture the structure of the catalyst was maintained after several runs. Also, the catalyst was analyzed by FESEM after 5 consecutive usages, and as can be seen, the morphology of the nanocatalyst has been completely preserved (Fig. 5f).

To confirm the successful synthesis of the nanocatalyst and to verify the presence of all the essential elements in its structure, the Energy Dispersive X-ray (EDX) technique was employed. (Fig. 5g). The EDX image illustrates the successful synthesis of nanoparticles and the excellent dispersion of all key elements such as Fe, Si, O, N, C, V, and Cl within the structure of modified iron nanoparticles with CS.VO_3_.HCl. Additionally, mapping analysis confirmed the proportional presence of Fe, N, O, C, V, Cl, and Si in the structure of the nanocatalyst (Fig. 5h). Furthermore, vanadium is effectively positioned on the surface of nanoparticles modified with chitosan.

**Figure 6:**
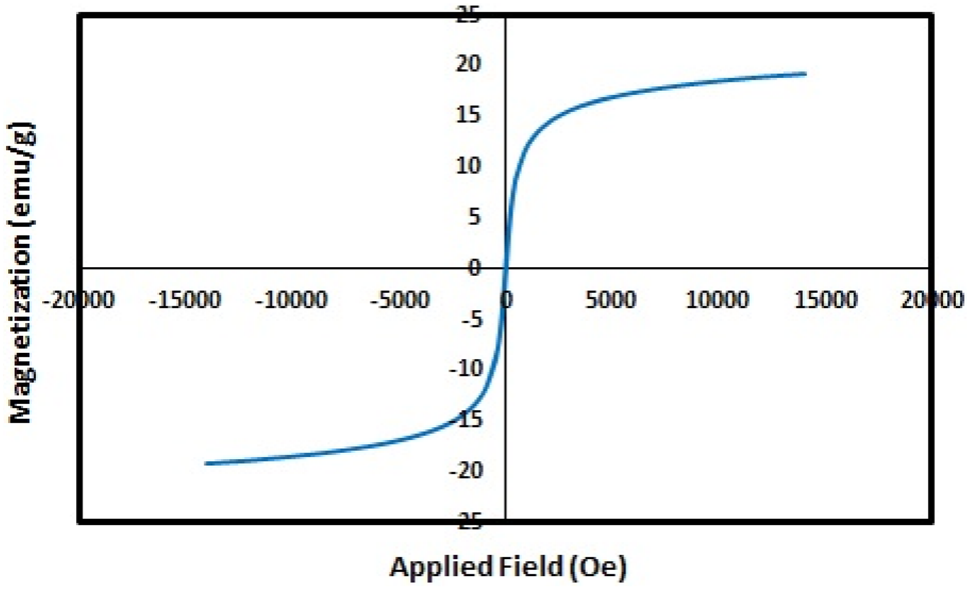
VSM curve for Fe_3_O_4_@SiO_2_@CSH^+^VO_3_^−^.

The VSM pattern of Fe_3_O_4_@SiO_2_@CSH^+^VO_3_^−^ was displayed in Fig. 6. The obtained magnetic strength is 20 emu/g, which due to covering the surface with TEOS and chitosan, the obtained magnetic strength indicates the easy separation of the nanocatalyst from the reaction mixture.

**Figure 7:**
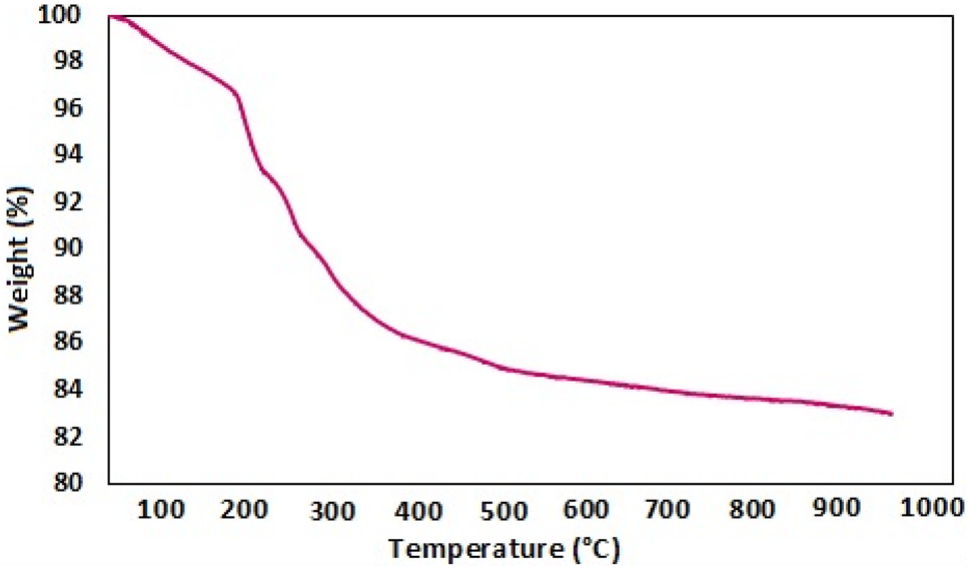
TGA diagram for Fe_3_O_4_@SiO_2_@CSH^+^VO_3_^−^

The thermal stability of the synthesized nanocatalyst was assessed using the TGA technique over a temperature range of 30–1000 °C. (Fig. 7). The TGA curve reveals several stages of weight reduction. Approximately 2% of weight loss is observed in the region of 30–200 °C, which can be attributed to the removal of organic solvents and moisture absorbed in the nanocatalyst structure^20,58^. A 10% weight loss, attributed to organic groups such as chitosan and inorganic groups such as vanadium attached to the surface of iron nanoparticles, occurs in the temperature range of 200–400 °C^59^. Moreover, within the temperature range of 400–1000 °C, a weight loss of 4% may indicate the decomposition of the silanized nanoparticles' structure.

**Figure 8:**
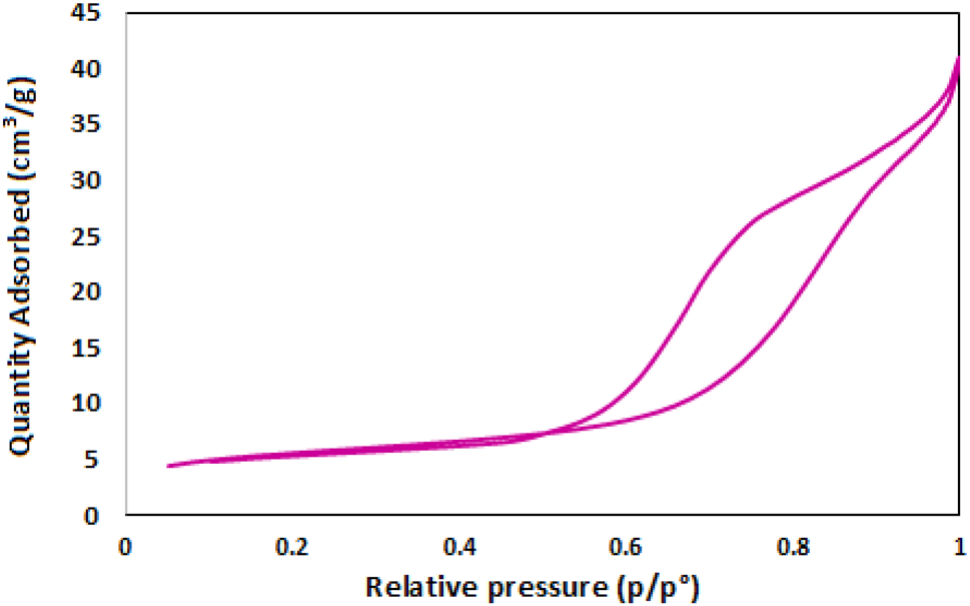
N_2_-adsorption–desorption plot of Fe_3_O_4_@SiO_2_@CSH^+^VO_3_^−^

Specific surface area (18.6324 ± 0.2385 m^2^/g), pore volume (0.060247 cm^3^/g) and pore size (129.3378 Å) were calculated by Brunauer–Emmett–Teller (BET) technique. According to the adsorption and desorption diagram, the synthesized nanocatalyst exhibits a type IV isotherm, indicative of the mesoporous structure of the nanoparticles (Fig. 8).

### Catalytic evaluation

After identifying and confirming the structure of the nanocatalyst introduced in this study, the catalytic efficiency of Fe_3_O_4_@SiO_2_@CSH^+^VO_3_^−^ in the preparation of sulfoxides was evaluated (Table 1). The reaction of benzyl phenyl sulfide with the oxidant (H_2_O_2_) was chosen as the selected reaction to optimize the reaction conditions. The impact of key variables such as the amount of catalyst, type of solvent, and amount of oxidant on the reaction process was thoroughly investigated. Initially, sulfide oxidation was examined in the absence of catalyst and oxidant, resulting in no sulfoxide formation. Furthermore, the reaction was conducted with the model in the presence of catalyst defects and with the oxidant in ethanol solvent, resulting in approximately 20% product formation. The effects of varying the amount of catalyst and the amount of hydrogen peroxide on the product percentage in ethanol solvent were then studied. First, the amount of oxidant was optimized. Amounts of 2–4 mmol of H_2_O_2_ were examined, and the best results were observed with 4 mmol of H_2_O_2_ (Table 1, entry 13). To optimize the amount of catalyst, 0.025 to 0.07 g of magnetic nanocatalyst were checked (Table 1, entries 10–14). Based on the results, the best efficiency (98%) was obtained in the presence of 0.4 mL of hydrogen peroxide and 0.05 g of catalyst in ethanol solvent and in 1 h (Table 1, entry 13). Next, the synergistic effect of different parts of the catalyst and the effect of the structure on the oxidation of sulfide were investigated. As can be deduced from the Table 1, entries 21–23, synergistic effects and morphology are not effective on the sulfide oxidation. The morphology of different parts is almost spherical (according to FESEM results). Sulfide was oxidized (98%) only in the presence of the final catalyst Fe_3_O_4_@SiO_2_@CSH^+^VO_3_^−^.

**Table 1:** Achieving the best reaction conditions for the oxidation of benzyl phenyl sulfide in the presence of Fe_3_O_4_@SiO_2_@CSH^+^VO_3_^−^.

Entry	Oxidant (mmol)	Catalyst (g)	Time (h)/T (°C)	Solvent	Sulfoxide (%)^a^
1	–	–	12/RT	EtOH	0
2	2	–	1/RT	EtOH	20
3	2	0.025	1/RT	EtOH	50
4	2	0.04	1/RT	EtOH	70
5	2	0.05	1/RT	EtOH	80
6	2	0.07	1/RT	EtOH	86
7	3	0.025	1/RT	EtOH	45
8	3	0.04	1/RT	EtOH	65
9	3	0.05	1/RT	EtOH	85
10	3	0.07	1/RT	EtOH	90
11	4	0.025	1/RT	EtOH	60
12	4	0.04	1/RT	EtOH	75
13	4	0.05	1/RT	EtOH	98
14	4	0.07	1/RT	EtOH	98
15	4	0.05	1/RT	CH_3_CN	75
16	4	0.05	1/RT	H_2_O	60
17	4	0.05	1/RT	DMF	88
18	4	0.05	1/RT	Toluene	40
19	4	0.05	1/RT	CHCl_3_	55
20	4	0.05	1/RT	–	65
21	4	0.05^b^	1/RT	EtOH	10
22	4	0.05^c^	1/RT	EtOH	15
23	4	0.05^d^	1/RT	EtOH	20

Reaction conditions: Benzyl phenyl sulfide (1 mmol) H_2_O_2_ and Fe_3_O_4_@SiO_2_@CSH^+^VO_3_^−^, in ethanol and 25 °C.

^a^Yields: Isolated yields.

^b^Cat: Fe_3_O_4_ NPs.

^c^Cat: Fe_3_O_4_@SiO_2_.

^d^Cat_:_ Fe_3_O_4_@SiO_2_@CS.

After determining the optimal amount of oxidant and catalyst, the influence of solvent polarity on the extent of sulfide oxidation was examined. Solvents with varying polarity, including acetonitrile, water, DMF, toluene, chloroform, and solvent-less conditions, were evaluated. Interestingly, in all cases, the desired sulfide was oxidized with lower yields compared to ethanol solvent. Additionally, increasing the amount of H_2_O_2_ led to the exclusive formation of sulfoxide without the formation of sulfone product.

After obtaining the optimized conditions, various aromatic and aliphatic sulfides were oxidized in the presence of hydrogen peroxide and Fe_3_O_4_@SiO_2_@CSH^+^VO_3_^−^ in ethanol (Table 2). The presented catalyst exhibited remarkable performance for the oxidation of sulfides, with the desirable sulfoxide prepared with high efficiency and in a relatively short time in all cases. It is noteworthy that aromatic sulfides containing electron-withdrawing groups yielded products in longer reaction times and with lower efficiency compared to aromatic sulfides with electron-donating groups. Additionally, the method demonstrated exceptional chemoselectivity in the oxidation of sulfides, where even in the presence of sensitive alcohol groups, only the sulfide was oxidized while the alcohol group remained intact. Furthermore, 2-(benzylthio)-1*H*-benzimidazole, as a heterocyclic sulfide, produced the desired sulfoxide with excellent yield.

**Table 2:** Oxidation of sulfides into sulfoxides catalyzed by Fe_3_O_4_@SiO_2_@CSH^+^VO_3_^−^.

Entry	Sulfoxide	Time (h)	Yield (%)	Mp^Ref^ (°C)
1		1.5	93	29–30^35^
2		1	95	69–71^35^
3		1	98	121–123^35^
4		1.5	95	122–124^60^
5		2	94	141–143^61^
6		2	94	137^35^
7		1.5	92	131–133^61^
8		2	92	162–164^35^
9		2.5	94	171–173^35^
10		2.5	92	110–112^35^
11		2	94	150–152^35^
12		1.5	95	29–30^60^

Reaction conditions: sulfide (1 mmol), H_2_O_2_ (0.4 mL), Catalyst (0.05 g), EtOH, 25 °C.

**Figure 9:**
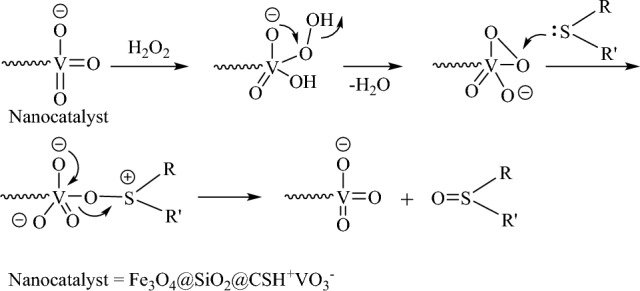
A possible mechanism for the oxidation of sulfides in the presence of Fe_3_O_4_@SiO_2_@CSH^+^VO_3_^−^

The details of sulfide oxidation in the presence of Fe_3_O_4_@SiO_2_@CSH^+^VO_3_^−^ are as follows (Fig. 9). In the initial step, H_2_O_2_ can bind to vanadium on the catalyst surface, resulting in the removal of one molecule of water. Subsequently, the sulfide, acting as a nucleophile, attaches to the oxygen atom bound to the vanadium, ultimately leading to the formation of the sulfoxide^62^.

### Reusability of the Fe_3_O_4_@SiO_2_@CSH^+^VO_3_^−^

**Figure 10:**
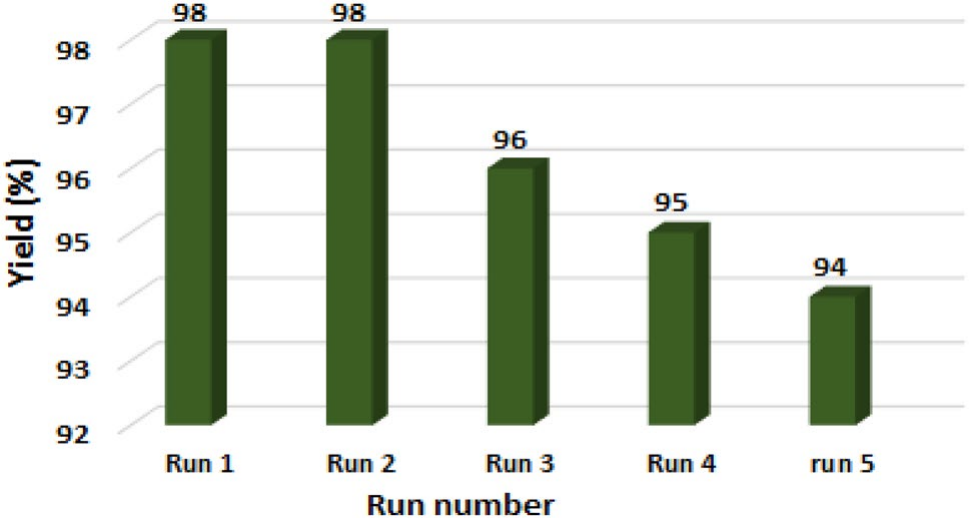
Ability to recover the catalyst up to at least 5 times for the model reaction.

One of the prominent objectives in green chemistry is the utilization of nanocatalysts that can be easily recovered from the reaction medium. Therefore, the recovery capability of the nanocatalyst in the sulfide oxidation reaction under optimized conditions was examined using a model reaction. After the formation of the sulfoxide, the catalyst was removed from the environment using a magnet, washed with water and ethanol, and then dried for subsequent reactions. As shown in Fig. 10, after 5 consecutive uses, a slight decrease in catalytic activity was observed, which could be attributed to contamination of the nanocatalyst surface.

**Figure 11:**
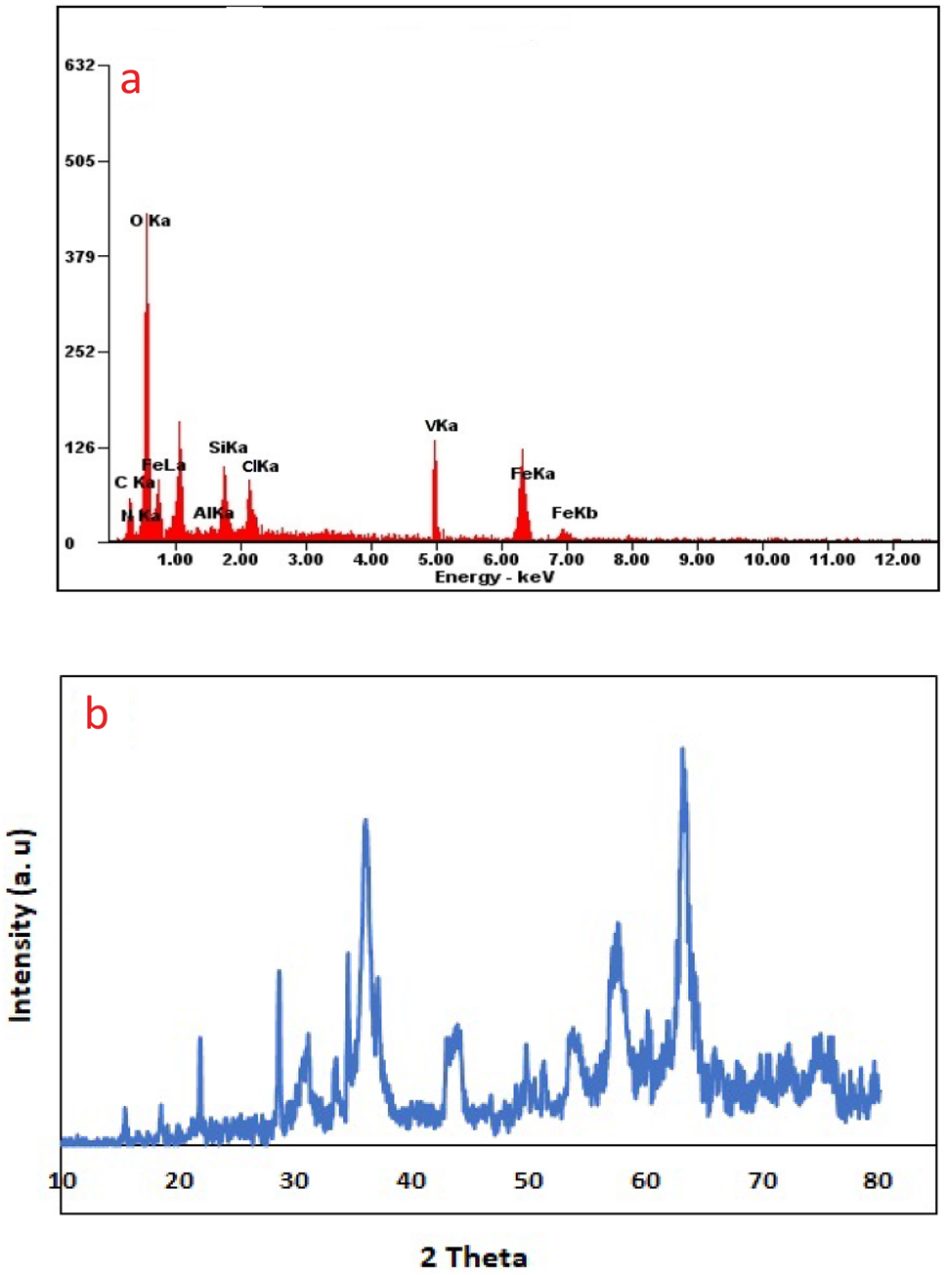
EDX pattern **(a)** and XRD curve **(b)** for catalyst after 5th run.

EDX and XRD analyses were performed on the nanocatalyst after 5 consecutive uses. It is noteworthy that all the main elements can be observed in the EDX Catalyst image after the fifth use (Fig. 11a). As shown in Fig. 11b, the structure of the nanocatalyst remains intact with no significant changes observed. In the XRD spectrum, all the elements are present, with only variations in the intensity of the peaks, either decreasing or increasing.

### Leaching test

Furthermore, a leaching test was conducted to confirm the heterogeneous nature of the prepared Fe_3_O_4_@SiO_2_@CSH^+^VO_3_^−^ catalyst for the oxidation of sulfides. In this test, benzyl phenyl sulfide was chosen as the selected reaction under optimal conditions. Halfway through the reaction time (30 min), the reaction was halted (yielding 51%), and the catalyst was separated from the reaction mixture using a magnet. Subsequently, the mixture was allowed to continue in the absence of the catalyst under stirring, and after a certain period, only a negligible increase of about 2% in the yield of sulfoxide was observed. This minimal change in the product quantity confirms the heterogeneous nature of the catalyst and indicates the absence of vanadium leaching into the reaction medium.

### Comparison of catalyst efficiency

To compare the efficiency of the introduced nanocatalyst, several scientific reports were reviewed, all of which involve iron nanoparticles modified with different metals or linkers. The results are summarized in Table 3. As shown in Table 3, the synthesized magnetic nanocatalyst in this study exhibits superiority over other catalytic systems in terms of reaction time and efficiency. Additionally, it is comparable in terms of the amount of oxidant and solvent used. Specifically, the oxidation of benzyl phenyl sulfide was compared.

**Table 3:** Comparison of the performance of the synthesized magnetic nanocatalyst with several other catalytic systems.

Entry	Conditions	Catalyst	Time (h)	Yield^Ref^
1	H_2_O_2_ (3 mmol), 40 ℃, CH_3_CN	Fe_3_O_4_/CS/HWO (5 mmol)	3	75^63^
2	H_2_O_2_ (10 mmol), 40 ℃, solvent-free	VOPDA/Fe_3_O_4_ (1.25 mol%)	1.1	96^64^
3	H_2_O_2_ (5 mmol), 25 ℃, solvent-free	Fe_3_O_4_/AMPD/Pd	0.5	92^65^
4	H_2_O_2_ (15 mmol), 25 ℃, CH_3_CN	Fe_3_O_4_-DETA-SA (10 mol%)	1	94^66^
5	H_2_O_2_ (4 mmol), 25 ℃, H_2_O	Fe_3_O_4_@SiO_2_-FeQ_3_ (10 mol%)	1.5	94.2^67^
6	H_2_O_2_ (4 mmol), 25 ℃, solvent-free	Fe_3_O_4_@CS-Cu complex (0.05 g)	10	93^68^
7	H_2_O_2_ (4 mmol), 25 ℃, EtOH	Fe_3_O_4_@SiO_2_-APTES(Fe(acac)_2_) (10 mol%	2.7	91^69^
8	H_2_O_2_ (4 mmol), 25 ℃, solvent-free-(EtOH for solid sulfides)	Fe_3_O_4_@Chitosan-Bound Picolinaldehyde Cu complex (0.05 g)	22	93^70^
9	H_2_O_2_ (4 mmol), 25 ℃, EtOH	This work	1	98

## Conclusion

In summary, this research presents the synthesis of a novel heterogeneous magnetic nanocatalyst containing a vanadium-chitosan complex through a simple and cost-effective method. The nanocatalyst exhibited selective conversion of sulfides into sulfoxides under mild conditions with high yield. Notably, minimal leaching of vanadium from the catalyst surface was observed, which is environmentally beneficial. Comprehensive analyses including IR, XRD, TEM, FESEM, EDX, mapping, TGA, and VSM confirmed the successful synthesis of the nanocatalyst. This procedure offers several advantages such as easy catalyst preparation, no requirement for special or harsh conditions, use of inexpensive and readily available raw materials, facile catalyst recovery, simple separation from the reaction mixture, excellent activity and selectivity, and the potential for catalyst reuse without significant loss in catalytic efficacy.

### Supplementary Information

Supplementary Information.

## Data Availability

Data from this research are available upon sensible request from the corresponding author.
