# Supplementary material for: Fe3O4@SiO2@CSH+VO3− as a novel recyclable heterogeneous catalyst with core–shell structure for oxidation of sulfides
Source: Sci Rep. 2024 Apr 8;14:8175. doi: 10.1038/s41598-024-58552-3 (PMC11001875; doi:10.1038/s41598-024-58552-3)

Supplementary Information for Scientific Reports journal

[**Fe_3_O_4_@SiO_2_@CSH^+^VO_3_**](mailto:Fe3O4@SiO2@CS.HCl.VO3)**^-^ as A Novel Recyclable Heterogeneous Catalyst with Core-Shell structure for Oxidation of Sulfides**

**Ulla Zubaidi,^1^ Kiumars Bahrami,^1,2*,^Minoo Khodamorady^1^**

^1^Department of Organic Chemistry, Faculty of Chemistry, Razi University, Kermanshah 67144-14971, Iran.

^2^Nanoscience and Nanotechnology Research Center (NNRC), Razi University, Kermanshah 67144-14971, Iran. *email: kbahrami2@hotmail.com; [k.bahrami@razi.ac.ir](mailto:k.bahrami@razi.ac.ir).

| Content | Page |
| --- | --- |
| Selected ^1^H NMR and ^13^C NMR of products | 2-4 |

Table 2, entry 3


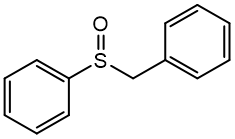

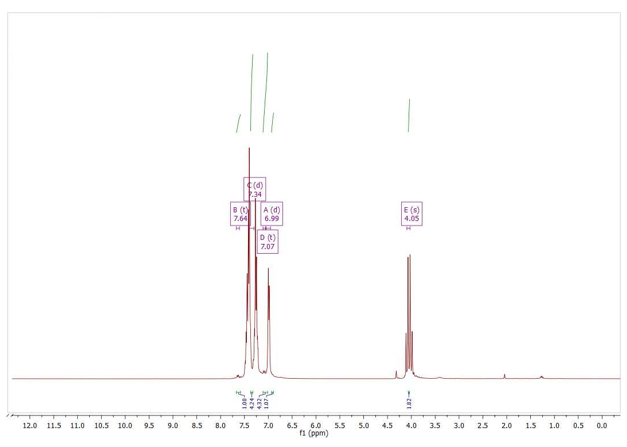


Table 2, entry 3


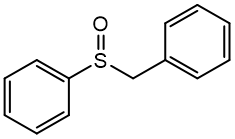


Table 2, entry 5


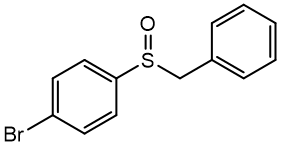

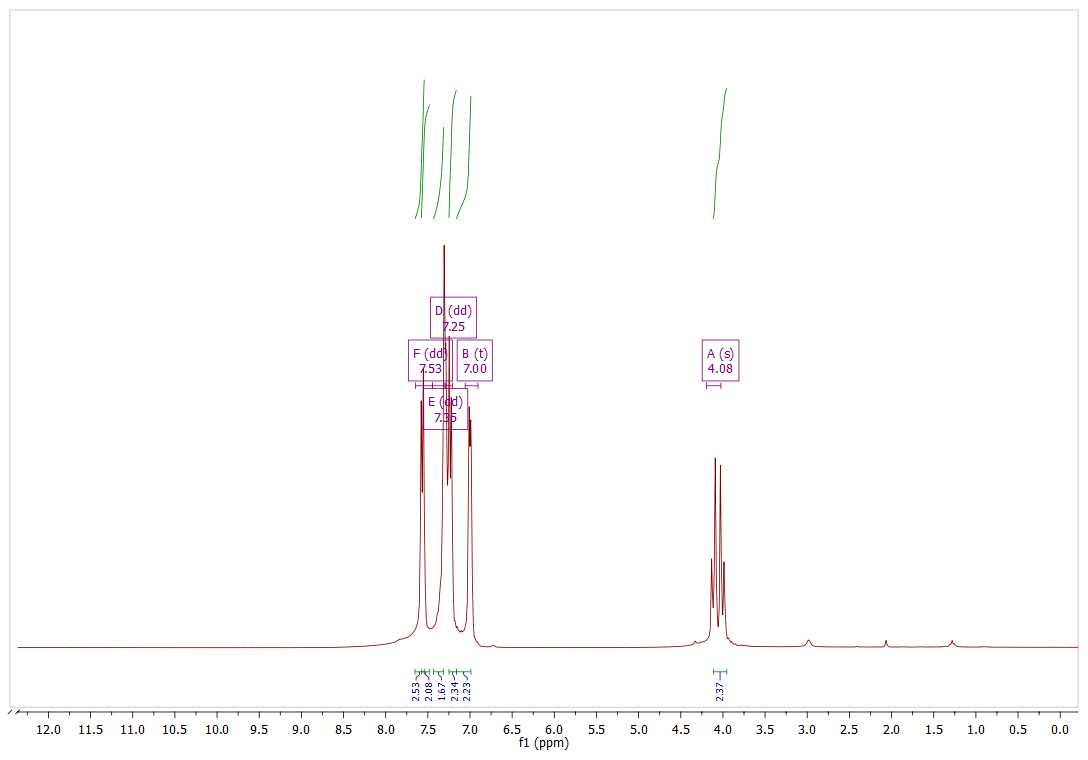


Table 2, entry 5


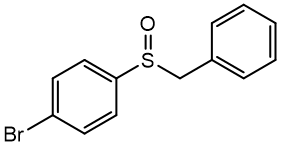

Supplement: Supplementary file 1 — Supplementary Information. [file 41598_2024_58552_MOESM1_ESM.docx]
